# Author Correction: Cellular census of human fibrosis defines functionally distinct stromal cell types and states

**DOI:** 10.1038/s41467-020-17073-z

**Published:** 2020-06-24

**Authors:** Thomas B. Layton, Lynn Williams, Fiona McCann, Mingjun Zhang, Marco Fritzsche, Huw Colin-York, Marisa Cabrita, Michael T. H. Ng, Marc Feldmann, Stephen N. Sansom, Dominic Furniss, Weilin Xie, Jagdeep Nanchahal

**Affiliations:** 1 The Kennedy Institute of Rheumatology, Nuffield Department of Orthopaedics, Rheumatology and Musculoskeletal Sciences, University of Oxford, Oxford, UK; 2 Department of Inflammation Research, Celgene Corporation, San Diego, CA USA; 3 MRC Human Immunology Unit, Weatherall Institute of Molecular Medicine, University of Oxford, Headley Way, Oxford, UK; 4 Nuffield Department of Orthopaedics, Rheumatology, and Musculoskeletal Sciences, University of Oxford, Botnar Research Centre, Oxford, UK; 5 Present Address: Institute of Materia Medica, Shandong First Medical University & Shandong, Academy of Medical Sciences, Jinan, Shandong Province P. R. China

**Keywords:** Translational research, Molecular medicine

Correction to: *Nature Communications* 10.1038/s41467-020-16264-y, published online 2 June 2020.

The original version of this Article contained an error in the spelling of the authors Stephen N. Sansom and Marco Fritzsche, which were incorrectly given as Stephen Samson and Marco Fritszche. This has now been corrected in both the PDF and HTML versions of the Article.

