# Supplementary material for: Cellular census of human fibrosis defines functionally distinct stromal cell types and states
Source: Nat Commun. 2020 Jun 2;11:2768. doi: 10.1038/s41467-020-16264-y (PMC7265502; doi:10.1038/s41467-020-16264-y)
Supplement: Supplementary file 1 — Supplementary Information [file 41467_2020_16264_MOESM1_ESM.pdf]

Cellular census of human fibrosis defines functionally distinct stromal cell types and states

Authors

Thomas B Layton[1], Lynn Williams[1], Fiona McCann[1], Mingjun Zhang[2], Marco Fritszche[1], Huw Colin-York[3], Marisa Cabrita[1], Michael T H Ng[3], Marc Feldmann[1], Stephen Samson[1], Dominic Furniss[4], Weilin Xie[2],[5] , Jagdeep Nanchahal[1†]

Affiliations

[1]The Kennedy Institute of Rheumatology, Nuffield Department of Orthopaedics, Rheumatology and Musculoskeletal Sciences, University of Oxford, UK

[2]Department of Inflammation Research, Celgene Corporation, San Diego, California, United States of America.

[3]MRC Human Immunology Unit, Weatherall Institute of Molecular Medicine, University of Oxford, Headley Way, Oxford OX3 9DS, UK.

[4]Nuffield Department of Orthopaedics, Rheumatology, and Musculoskeletal Sciences, University of Oxford, Botnar Research Centre, UK.

[5]Present address: Institute of Materia Medica, Shandong First Medical University & Shandong, Academy of Medical Sciences, Jinan, Shandong Province, P R China

†Corresponding author. Email: jagdeep.nanchahal@kennedy.ox.ac.uk

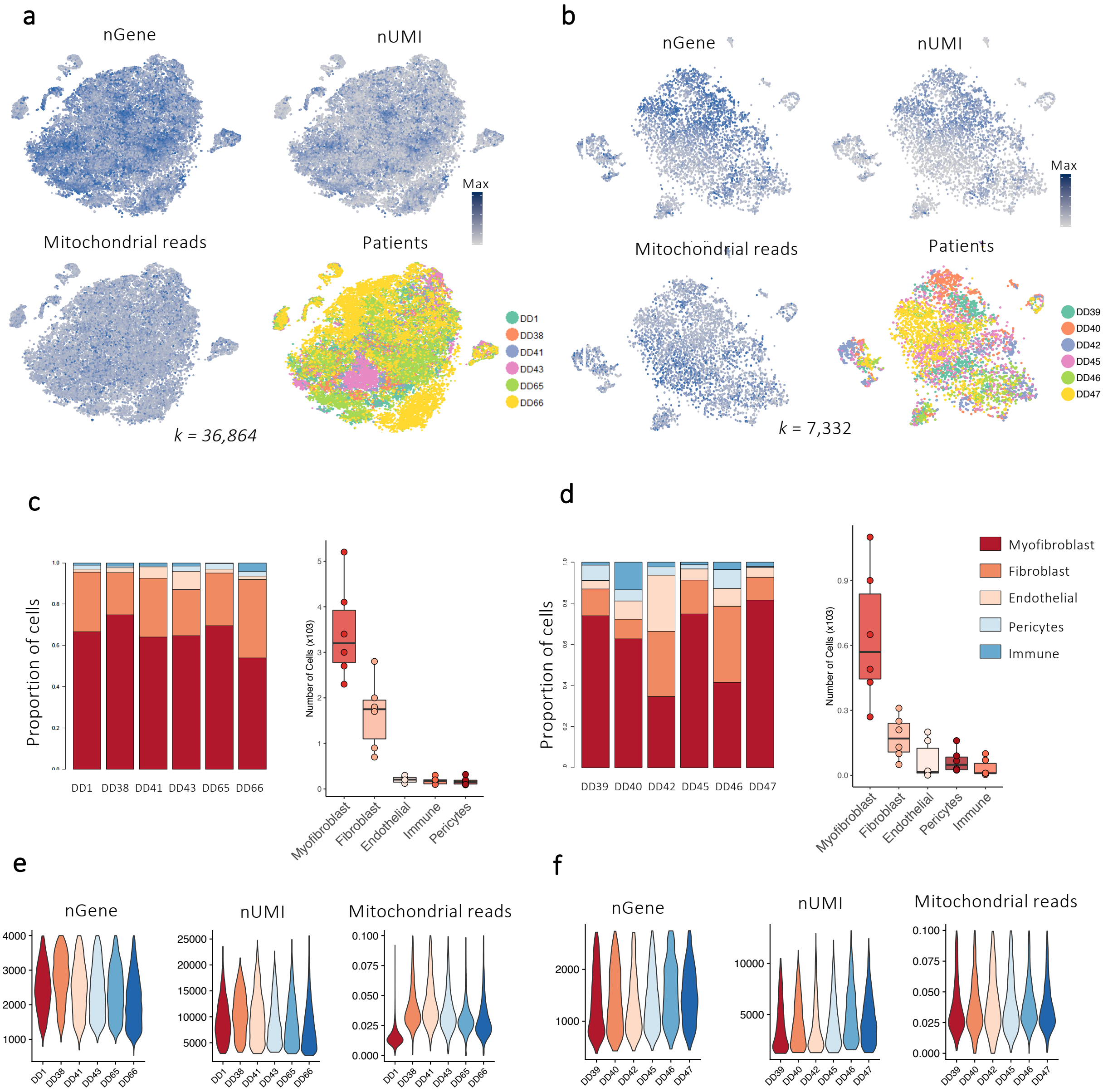
a
nGene
nUMI
Max
Mitochondrial reads
Patients
DD1
DD38
DD41
DD43
DD65
DD66
k = 36,864
b
nGene
nUMI
Max
Mitochondrial reads
Patients
DD39
DD40
DD42
DD45
DD46
DD47
k = 7,332
c
Proportion of cells
DD1 DD38 DD41 DD43 DD65 DD66
Number of Cells (x103)
Myofibroblast
Fibroblast
Endothelial
Immune
Pericytes
d
Proportion of cells
DD39 DD40 DD42 DD45 DD46 DD47
Number of Cells (x103)
Myofibroblast
Fibroblast
Endothelial
Pericytes
Immune
e
nGene
nUMI
Mitochondrial reads
f
nGene
nUMI
Mitochondrial reads

Supplementary Figure 1. **Quality control for single cell RNA-seq of Dupuytren's nodules**

a-**b**, tSNE projections showing the number of genes and UMIs, percentage of mitochondrial reads and patient donors for 36,864 cells from the first patient batch (a) (*n*= 6 DD patients) and 7,332 cells from the second (b) patient batch (*n* = 6 DD patients) in single cell RNA-seq. Coloured by z-score values of QC metrics. **c**-**d**, Bar plots and box and whisker plots showing the proportion and number of cell types in first (c) and second (d) six patients in single cell RNA-seq. **e-f**, For box and whisker plots, range 90-5200 cells, median 280 cells and box bounds 155-1950 representing first and third quantiles. Violin plots showing quality control metrics in first (e) and second six patients (f) in single cell RNA-seq. Y axis in log(UMI+1) for nGene and nUMI and percentage of cells for mitochondrial reads. DD is prefix for DD patient IDs (*n* = 12 DD patients).

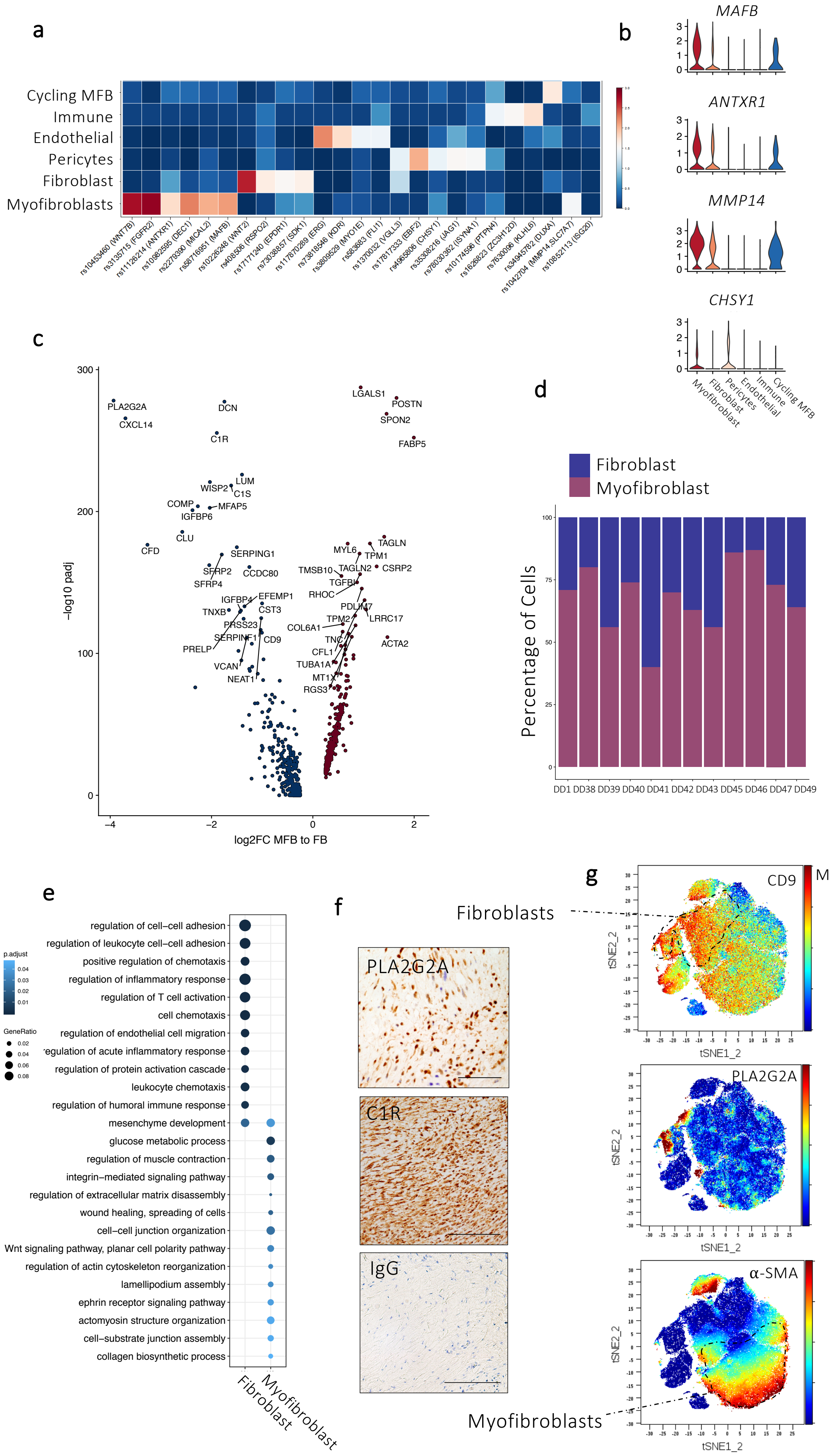

a
Cycling MFB
Immune
Endothelial
Pericytes
Fibroblast
Myofibroblasts
rs10453460 (WNT7B)
rs3135715 (FGFR2)
rs11126214 (ANTXR1)
rs10982595 (DEC1)
rs2279390 (MICAL2)
rs58716951 (MAFB)
rs10226248 (WNT2)
rs408506 (RSPO2)
rs17171240 (EPDR1)
rs73038857 (SDK1)
rs117870289 (ERG)
rs73818546 (KDR)
rs3809529 (MYO1E)
rs583683 (FLI1)
rs1370032 (VGLL3)
rs17817333 (EBF2)
rs4965806 (CHSY1)
rs35308216 (JAG1)
rs78030362 (ISYNA1)
rs10174596 (PTPN4)
rs1626823 (ZC3H12D)
rs7630096 (KLHL6)
rs34945782 (DUXA)
rs1042704 (MMP14,SLC7A7)
rs10852113 (ISG20)
b
MAFB
ANTXR1
MMP14
CHSY1
Myofibroblast
Fibroblast
Pericytes
Endothelial
Immune
Cycling MFB
c
PLA2G2A
CXCL14
DCN
C1R
LGALS1
POSTN
SPON2
FABP5
LUM
WISP2
C1S
COMP
MFAP5
IGFBP6
CLU
CFD
SERPING1
SFRP2
SFRP4
CCDC80
IGFBP4
EFEMP1
TNXB
CST3
PRSS23
SERPINF1
CD9
PRELP
VCAN
NEAT1
MYL6
TAGLN
TPM1
TMSB10
TAGLN2
CSRP2
TGFBI
RHOC
PDLIM7
TPM2
LRRC17
COL6A1
TNC
ACTA2
CFL1
TUBA1A
MT1X
RGS3
−log10 padj
log2FC MFB to FB
d
Fibroblast
Myofibroblast
Percentage of Cells
DD1 DD38 DD39 DD40 DD41 DD42 DD43 DD45 DD46 DD47 DD49
e
p.adjust
GeneRatio
regulation of cell−cell adhesion
regulation of leukocyte cell−cell adhesion
positive regulation of chemotaxis
regulation of inflammatory response
regulation of T cell activation
cell chemotaxis
regulation of endothelial cell migration
regulation of acute inflammatory response
regulation of protein activation cascade
leukocyte chemotaxis
regulation of humoral immune response
mesenchyme development
glucose metabolic process
regulation of muscle contraction
integrin−mediated signaling pathway
regulation of extracellular matrix disassembly
wound healing, spreading of cells
cell−cell junction organization
Wnt signaling pathway, planar cell polarity pathway
regulation of actin cytoskeleton reorganization
lamellipodium assembly
ephrin receptor signaling pathway
actomyosin structure organization
cell−substrate junction assembly
collagen biosynthetic process
Fibroblast
Myofibroblast
f
PLA2G2A
C1R
IgG
g
Fibroblasts
CD9
Max
PLA2G2A
α-SMA
Myofibroblasts
tSNE1_2
tSNE2_2

Supplementary Figure 2. **Fibroblasts and myofibroblast have distinct signatures in human fibrosis.**

a, Heatmap showing specificity of DD-associated GWAS loci in immune and mesenchymal cell populations in single cell RNA-seq of DD nodules using SNPSea. Heatmap represents z-score expression of DD GWAS loci associated genes (*n*=12 DD patients). **b**, Violin plots showing expression of DD GWAS associated genes in scaled log(UMI+1) in cell types in Dupuytren's nodules. **c**, Volcano plot showing differentially expressed genes (2-sided Wilcoxon Rank Sum Test) between myofibroblast and fibroblasts in single cell RNA-seq with FDR corrected adjusted *p*-values (BH correction). MFB = myofibroblast and FB = fibroblasts. **d**, Stacked bar plot showing the proportion of myofibroblasts to fibroblasts in each Dupuytren's nodule (*n* = 12 DD patients, DD is prefix for patient ID). **e**, Dot plot showing pathways enriched in fibroblasts and myofibroblasts in single cell RNA-seq. Gene ratio is the number of genes found in pathways and p.adjust is adjusted p-value (2-sided Wilicoxon Rank Sum test, BH FDR-correction). **f**, Microscopy images of Immunohistochemistry showing fibroblast marker expression in Dupuytren's nodules with isotype control (Rabbit IgG) (*n* = 5 DD patients). Scale bar = 20 µm. **g**, tSNE projections of CyTOF analysis for representative DD patient showing fibroblast markers CD9 and PLA2G2A, alongside myofibroblast marker α-SMA (*n* = 6 DD patients). Scale bar is normalized protein expresion.

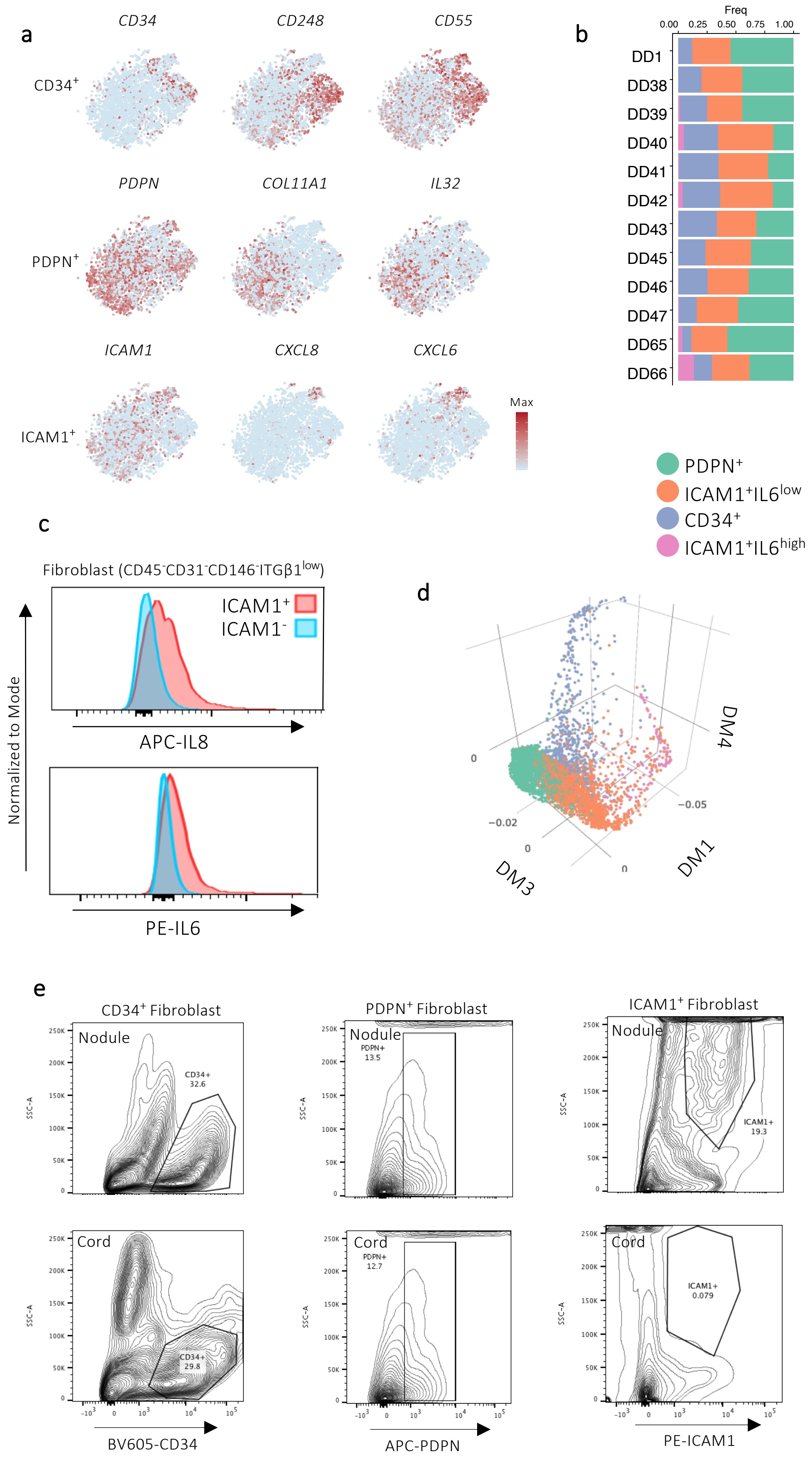

a
CD34
CD248
CD55
CD34+
PDPN
COL11A1
IL32
PDPN+
ICAM1
CXCL8
CXCL6
ICAM1+
Max
b
Freq
0.00 0.25 0.50 0.75 1.00
DD1
DD38
DD39
DD40
DD41
DD42
DD43
DD45
DD46
DD47
DD65
DD66
PDPN+
ICAM1+IL6low
CD34+
ICAM1+IL6high
c
Fibroblast (CD45-CD31-CD146-ITGβ1low)
ICAM1+
ICAM1-
Normalized to Mode
APC-IL8
PE-IL6
d
DM4
DM3
DM1
e
CD34+ Fibroblast
PDPN+ Fibroblast
ICAM1+ Fibroblast
Nodule
CD34+ 32.6
PDPN+ 13.5
ICAM1+ 19.3
Cord
CD34+ 29.8
PDPN+ 12.7
ICAM1+ 0.079
SSC-A
BV605-CD34
APC-PDPN
PE-ICAM1

Supplementary Figure 3. **Three major subsets of fibroblasts in fibrosis housing an chemokine-rich ICAM1$^+$ population.**

a, tSNE projections of single cell RNA-seq of fibroblasts coloured by z-score expression of marker genes of major subsets (*CD34*, *PDPN* & *ICAM1*). (*n*=12 DD patients). **b**, Bar plot showing the proportion of fibroblast subsets in 12 Dupuytren's patients as a total of the entire fibroblast population in single cell RNA-seq. DD is patient ID prefix (*n*=12 DD patients). **c**, Histograms of flow cytometry analysis of freshly isolated and unstimulated nodular cells showing IL-6 and IL-8 protein expression in ICAM1^{+}/$^{-}$ fibroblasts (CD31$^-$CD45$^-$CD146$^-$ITG-β1^{low}). (*n*=3 DD patients). **d**, Diffusion map projection of single cell RNA-seq of fibroblasts coloured by subsets. DM1, 3 and 4 are diffusion component coordinates (*n*= 12 DD patients). **e***,* Representative density plots of flow cytometry analysis for three fibroblast populations (PDPN$^+$, CD34$^+$ & ICAM1$^+$) in the myofibroblast and immune cell-rich nodule and matrix-rich cord. (*n*= 8 DD patients).

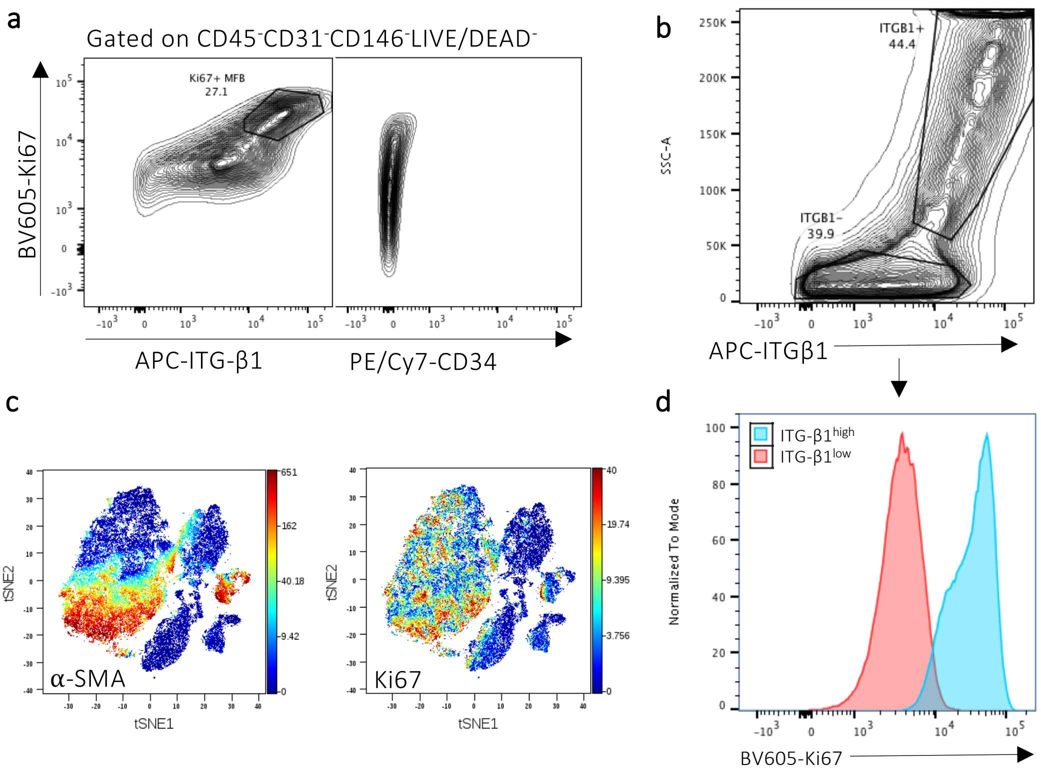
a
Gated on CD45-CD31-CD146-LIVE/DEAD-
Ki67+ MFB
27.1
BV605-Ki67
APC-ITG-β1
PE/Cy7-CD34
b
ITGB1+
44.4
ITGB1-
39.9
SSC-A
APC-ITGβ1
c
α-SMA
Ki67
tSNE1
tSNE2
d
ITG-β1high
ITG-β1low
Normalized To Mode
BV605-Ki67

Supplementary Figure 4. **The proliferative stromal cell is housed within the myofibroblast compartment.**

a, Density plots of flow cytometry analysis for freshly isolated nodular cells showing Ki67 protein expression with myofibroblast marker ITG-β1 and fibroblast marker CD34. MFB = myofibroblast (*n* = 8 DD patients). **b**, Above, representative density plot of gating strategy for ITG-β1^{high} and ITG-β1^{low} stromal cell populations. **c**, tSNE projections of CyTOF analysis for representative DD patient showing Ki67^{+} enrichment in α-SMA^{+} myofibroblast population (*n* = 6 DD patients). **d**, Histogram showing FACS analysis of Ki67 expression in gated ITG-β1^{high} and ITG-β1^{low} populations (*n* = 6 DD patients).

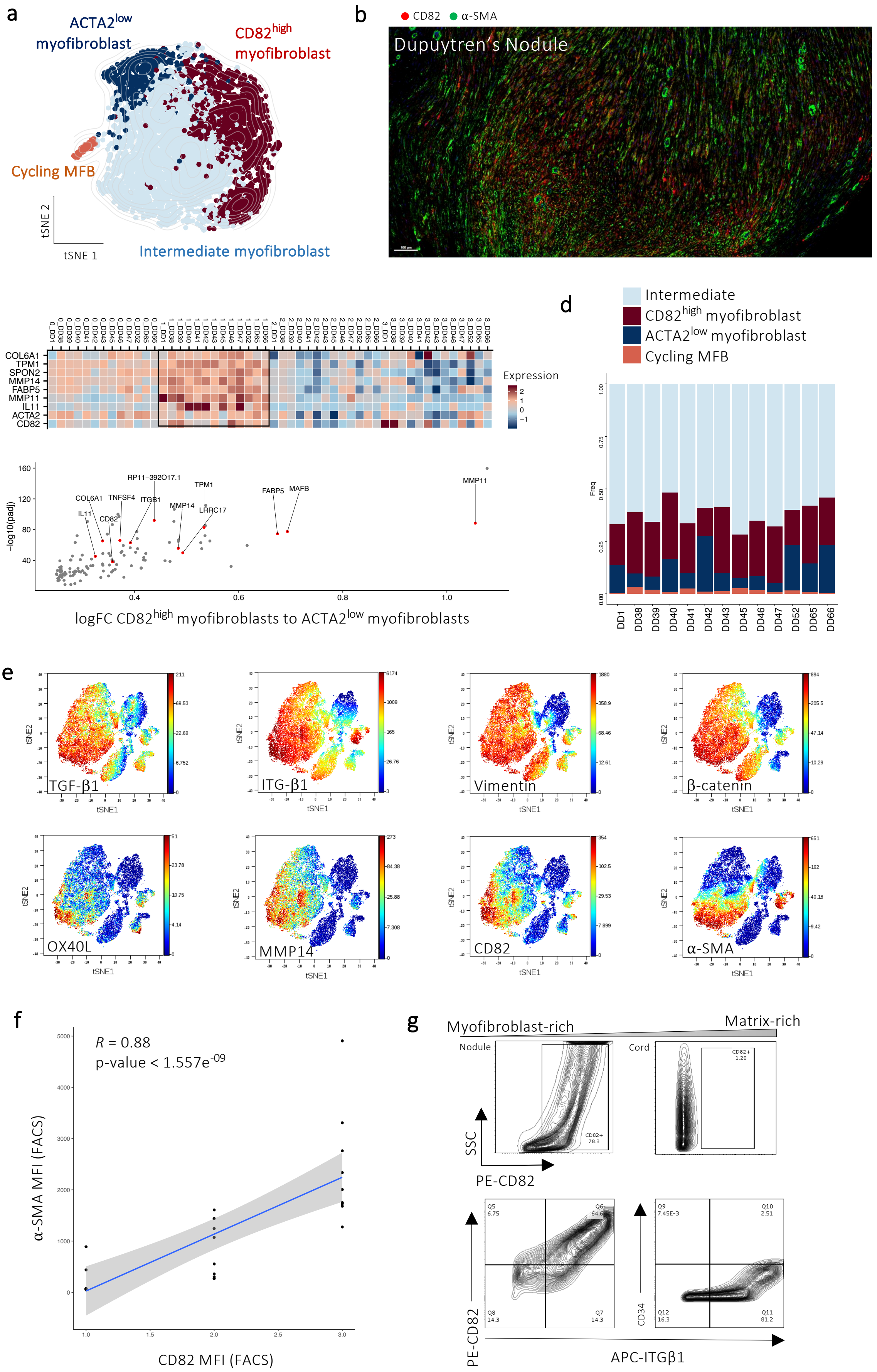
a
ACTA2low myofibroblast
CD82high myofibroblast
Cycling MFB
Intermediate myofibroblast
tSNE 1
tSNE 2
b
CD82
α-SMA
Dupuytren's Nodule
c
COL6A1
TPM1
SPON2
MMP14
FABP5
MMP11
IL11
ACTA2
CD82
Expression
logFC CD82high myofibroblasts to ACTA2low myofibroblasts
−log10(padj)
RP11−392O17.1
TPM1
FABP5
MAFB
MMP11
COL6A1
TNFSF4
ITGB1
MMP14
LRRC17
IL11
CD82
d
Intermediate
CD82high myofibroblast
ACTA2low myofibroblast
Cycling MFB
Freq
DD1
DD38
DD39
DD40
DD41
DD42
DD43
DD45
DD46
DD47
DD52
DD65
DD66
e
TGF-β1
ITG-β1
Vimentin
β-catenin
OX40L
MMP14
CD82
α-SMA
tSNE1
tSNE2
f
R = 0.88
p-value < 1.557e-09
α-SMA MFI (FACS)
CD82 MFI (FACS)
g
Myofibroblast-rich
Matrix-rich
Nodule
Cord
SSC
PE-CD82
PE-CD82
CD34
APC-ITGβ1

Supplementary Figure 5. Discrete CD82^{high} myofibroblast in localized human fibrosis.

a, tSNE projection of single cell RNA-seq showing four myofibroblasts subsets (*n* = 12 DD patients). **b**, Confocal image of immunofluorescence showing CD82 and α-SMA protein expression in Dupuytren's nodule (*n* = 5 DD patients). Scale bar 100 µm. **c**, Heatmap of single cell RNA-seq showing z-score mean expression of CD82^{high} myofibroblast markers (cluster 1) across DD patients. Prefix 0-3 denotes cluster and suffix DD1-DD65 represents DD patient IDs. Below, scatter plot showing top positive markers of CD82^{high} myofibroblasts. 2-sided Wilcoxon Rank Sum test and FDR-adjusted p-value (BH correction). p-adjust labelled < 1e^{-51} **d**, Bar plot of single cell RNA-seq showing the proportions of myofibroblast subsets in DD patients as a total of all myofibroblasts. (*n* = 12 DD patients). **e**, tSNE projections of CyTOF analysis showing distinct CD82^{high}OX40L^{+} myofibroblast in DD nodule. (*n = 6* DD patients). Scale bar is normalized protein expresion. **f**, Scatter plot with linear regression fit (mean +/- SE) of flow cytometry analysis showing positive correlation of CD82 and α-SMA protein expression. Data points represent mean fluorescence intensity (MFI) of α-SMA in CD82^{low}, CD82^{mid} and CD82^{high} myofibroblasts. These were selected as the top, middle and bottom 20% of cells based of CD82 expression (MFI), respectively. (*n* = 8 DD patients, *R* = Pearson correlation coefficient, 2-sided *t*-test statistics *p*-value = 1.557e^{-10}). **g**, Top, representative density plots of flow cytometry analysis showing increased CD82^{+} stromal cells in DD nodules as compared to DD cords (*n* = 6 DD patients). Bottom, density plot of flow cytometry analysis showing co-expression of α-SMA and ITG-β1 in myofibroblasts. CD34 used an example fibroblast marker.

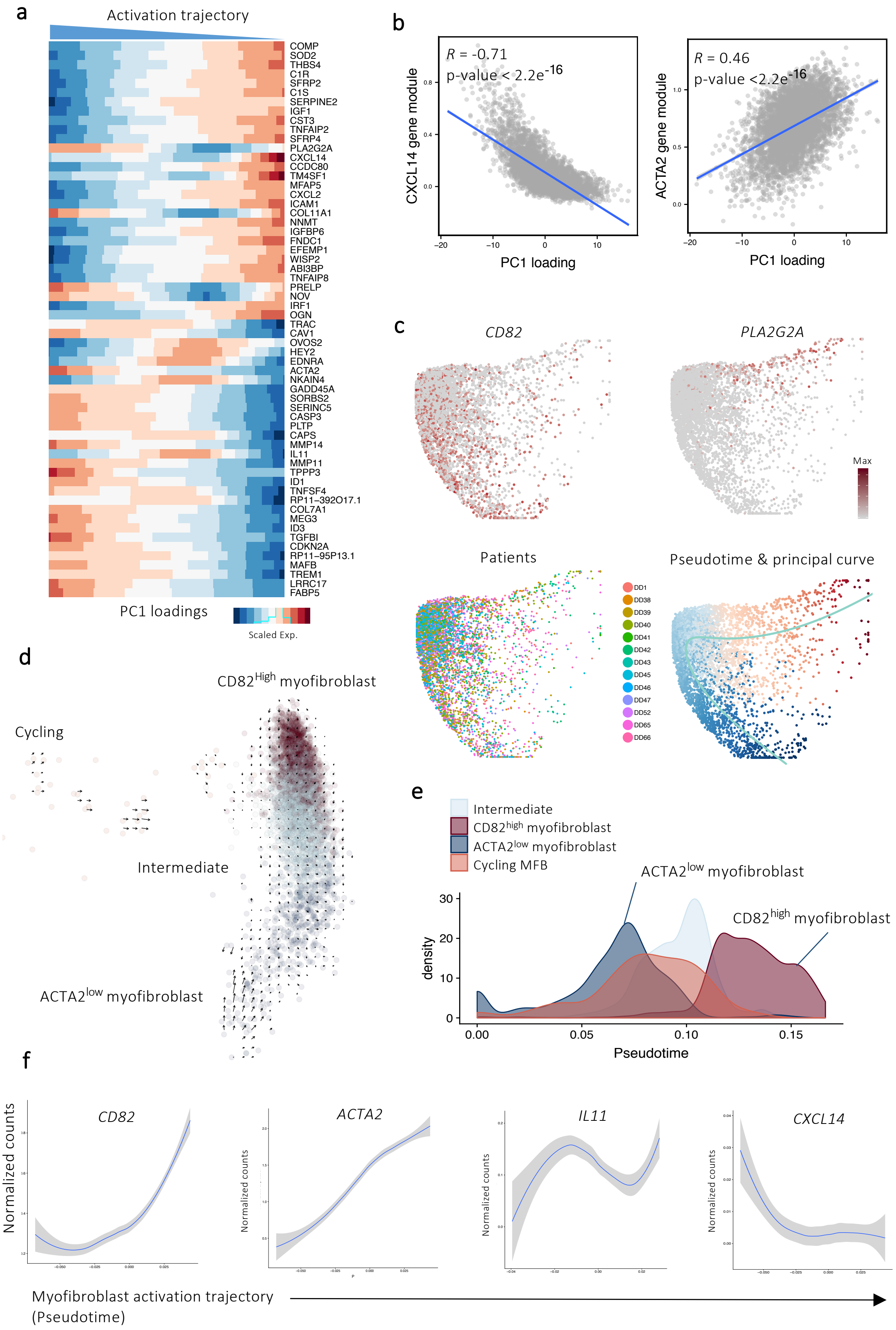
a
Activation trajectory
COMP
SOD2
THBS4
C1R
SFRP2
C1S
SERPINE2
IGF1
CST3
TNFAIP2
SFRP4
PLA2G2A
CXCL14
CCDC80
TM4SF1
MFAP5
CXCL2
ICAM1
COL11A1
NNMT
IGFBP6
FNDC1
EFEMP1
WISP2
ABI3BP
TNFAIP8
PRELP
NOV
IRF1
OGN
TRAC
CAV1
OVOS2
HEY2
EDNRA
ACTA2
NKAIN4
GADD45A
SORBS2
SERINC5
CASP3
PLTP
CAPS
MMP14
IL11
MMP11
TPPP3
ID1
TNFSF4
RP11−392O17.1
COL7A1
MEG3
ID3
TGFBI
CDKN2A
RP11−95P13.1
MAFB
TREM1
LRRC17
FABP5
PC1 loadings
Scaled Exp.
b
R = -0.71
p-value < 2.2e-16
CXCL14 gene module
PC1 loading
R = 0.46
p-value <2.2e-16
ACTA2 gene module
PC1 loading
c
CD82
PLA2G2A
Max
Patients
DD1
DD38
DD39
DD40
DD41
DD42
DD43
DD45
DD46
DD47
DD52
DD65
DD66
Pseudotime & principal curve
d
CD82High myofibroblast
Cycling
Intermediate
ACTA2low myofibroblast
e
Intermediate
CD82high myofibroblast
ACTA2low myofibroblast
Cycling MFB
ACTA2low myofibroblast
CD82high myofibroblast
density
Pseudotime
f
CD82
ACTA2
IL11
CXCL14
Normalized counts
Myofibroblast activation trajectory
(Pseudotime)

Supplementary Figure 6. **Modelling the myofibroblast activation trajectory associated with CD82 expression.**

a, Heatmap showing the first principal component (PC1) gene loadings (*loess* smoothed z-score row scaled expression) in single cell RNA-seq of myofibroblasts (*n*=12 DD patients). Putative myofibroblast activation trajectory direction labelled. **b**, Scatter plot of single cell RNA-seq with linear regression fit (mean +/- SE) of myofibroblast (*ACTA2* normalized expression*)* and fibroblast (*CXCL14* normalized expression*)* gene modules plotted against PC1 gene loadings. Each point represents a single myofibroblast (*n* = 12 DD patients). **c**, Diffusion map embedding of myofibroblasts coloured by the expression of CD82^{high} myofibroblast marker (*CD82*) and ACTA2^{low} (*PLA2G2A*) myofibroblast marker genes in scaled(log(UMI+1)). Bottom two figures are diffusion maps coloured by patient donors (*n*=12 DD patient*)* and slingshot pseudotime with overlay of principal curve fit (Spearman correlation with 2-sided *t*-test statistic). **d**, Representative PCA embedding of myofibroblasts coloured by four major subsets with overlying of RNA velocity. RNA velocity quantified using Velocyto (*n* = 12 DD patients) . **e**, Density plot of single cell RNA-seq of myofibroblasts illustrating subsets along pseudotime. Data represents Gaussian kernel probability densities. **f**, Line plots showing the expression (*loess* smoothed normalized expression +/- SE) of selected genes along modelled pseudotime (slingshot pseudotime) in single cell RNA-seq of myofibroblasts (*n* = 12 DD patients). Mean +/- SEM.

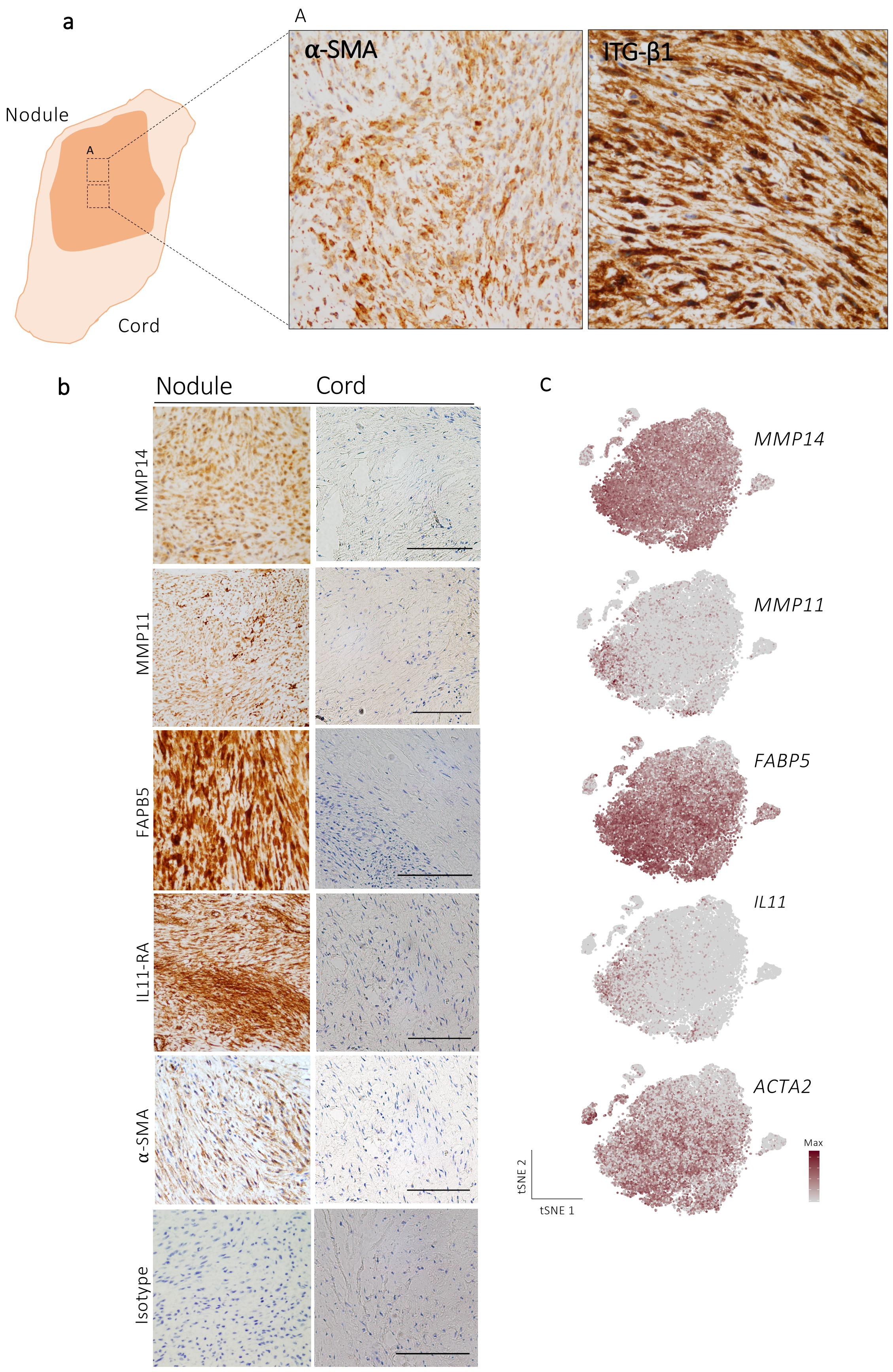
a
A
Nodule
A
Cord
α-SMA
ITG-β1
b
Nodule
Cord
MMP14
MMP11
FAPB5
IL11-RA
α-SMA
Isotype
c
MMP14
MMP11
FABP5
IL11
ACTA2
Max
tSNE 2
tSNE 1

Supplementary Figure 7. CD82^{high} myofibroblast markers are highly expressed within α-SMA rich foci *in vivo*.

a, Schematic of Dupuytren’s nodule and cord with representative immunohistochemistry staining for established myofibroblast markers. **b**, Microscopy image of immunohistochemistry staining in Dupuytren's nodule (myofibroblast and immune cell-rich fibrotic microenvironment) and cord (matrix-rich fibrotic microenvironment) for markers of CD82^{high} myofibroblast population (*n* = 5 DD patients). Scale bar 50 µm. **c**, tSNE projections of single cell RNA-seq coloured by the expression of corresponding CD82^{high} myofibroblast marker genes in z-score scaled log(UMI+1) (*n* = 6 DD patients). The IL11 receptor (IL11RA) used instead of IL11 given the lack of a suitable antibody.

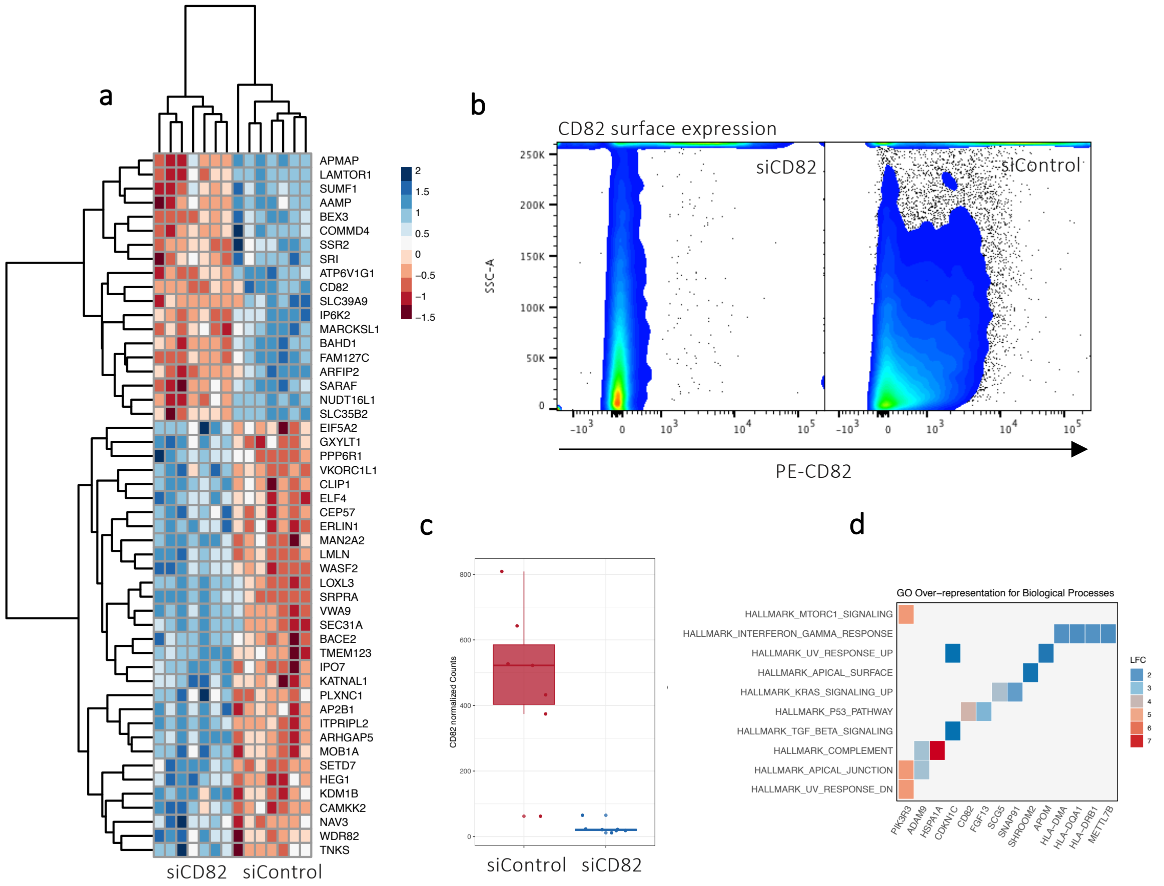

a
APMAP
LAMTOR1
SUMF1
AAMP
BEX3
COMMD4
SSR2
SRI
ATP6V1G1
CD82
SLC39A9
IP6K2
MARCKSL1
BAHD1
FAM127C
ARFIP2
SARAF
NUDT16L1
SLC35B2
EIF5A2
GXYLT1
PPP6R1
VKORC1L1
CLIP1
ELF4
CEP57
ERLIN1
MAN2A2
LMLN
WASF2
LOXL3
SRPRA
VWA9
SEC31A
BACE2
TMEM123
IPO7
KATNAL1
PLXNC1
AP2B1
ITPRIPL2
ARHGAP5
MOB1A
SETD7
HEG1
KDM1B
CAMKK2
NAV3
WDR82
TNKS
2
1.5
1
0.5
0
−0.5
−1
−1.5
siCD82 siControl
b
CD82 surface expression
siCD82
siControl
SSC-A
250K
200K
150K
100K
50K
0
-10^3
0
10^3
10^4
10^5
PE-CD82
c
CD82 normalized Counts
800
600
400
200
0
siControl siCD82
d
GO Over−representation for Biological Processes
HALLMARK_MTORC1_SIGNALING
HALLMARK_INTERFERON_GAMMA_RESPONSE
HALLMARK_UV_RESPONSE_UP
HALLMARK_APICAL_SURFACE
HALLMARK_KRAS_SIGNALING_UP
HALLMARK_P53_PATHWAY
HALLMARK_TGF_BETA_SIGNALING
HALLMARK_COMPLEMENT
HALLMARK_APICAL_JUNCTION
HALLMARK_UV_RESPONSE_DN
PIK3R3
ADAM9
HSPA1A
CDKN1C
CD82
FGF13
SCG5
SNAP91
SHROOM2
APOM
HLA−DMA
HLA−DQA1
HLA−DRB1
METTL7B
LFC
2
3
4
5
6
7

Supplementary Figure 8. **siRNA knockdown of tetraspanin CD82 in human myofibroblasts.**

a, Heatmap of z-score regularized-log transformed values of differentially expressed genes in RNA-seq following siCD82 and siControl transfection in DD myofibroblasts (*n* = 7 DD patients). **b**, Representative density plots of flow cytometry analysis of CD82 surface expression in DD myofibroblasts following siCD82 and siControl transfections (*n*=3 DD patients). **c**, Box and whisker plot of *CD82* normalized counts in RNA-seq following siCD82 and siControl transfections (*n*=7 DD patients, mean +/- SEM). Range 75-807, median 516, box bounds 401-594 represent first and third quantiles. **d**, Heatmap of RNA-seq data showing pathways enriched and contributing differentially expressed genes following siRNA mediated C*D82* knockdown. Colour scale represents $\log_2$ fold change. LFC = $\log_2$ fold change.

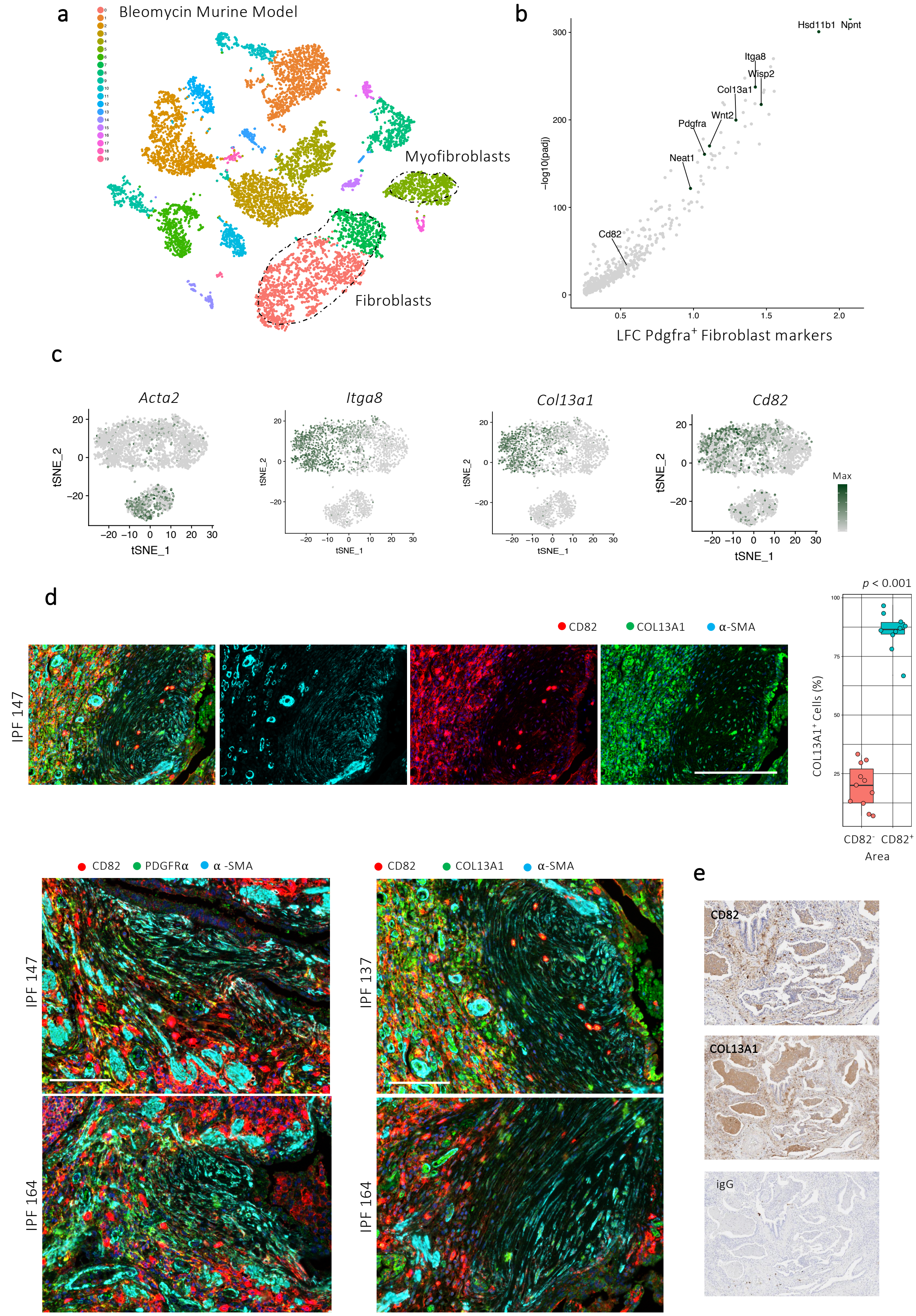

a
Bleomycin Murine Model
Myofibroblasts
Fibroblasts
b
Hsd11b1
Npnt
Itga8
Wisp2
Col13a1
Wnt2
Pdgfra
Neat1
Cd82
-log10(padj)
LFC Pdgfra+ Fibroblast markers
c
Acta2
Itga8
Col13a1
Cd82
tSNE_2
tSNE_1
Max
d
CD82
COL13A1
α-SMA
IPF 147
p < 0.001
COL13A1+ Cells (%)
CD82- CD82+
Area
CD82
PDGFRα
α -SMA
IPF 147
IPF 164
CD82
COL13A1
α-SMA
IPF 137
IPF 164
e
CD82
COL13A1
igG

Supplementary Figure 9. CD82$^+$PDGFRA$^+$ fibroblasts in murine and human pulmonary fibrosis.

a, tSNE projection of single cell RNA-seq of murine bleomycin pulmonary fibrosis model coloured by Louvain clusters with fibroblasts (*Col1a1$^+$Acta2$^-$*) and myofibroblasts (*Col1a1$^+$Acta2$^+$)* highlighted (*n* = 2 mice, *k* = 10,410 cells). **b**, Scatter plot showing significant marker genes (2-sided Wilcoxon Rank Sum Test) of Pdgfra$^+$ fibroblasts in single cell RNA-seq. LFC = log fold change. FDR-corrected (BH correction) p-value. **c**, tSNE projections of single cell RNA-seq of murine fibroblasts and myofibroblasts in bleomycin model (*n* = 2 mice) coloured by marker gene expression in scaled log(UMI+1). **d**, Confocal images of immunofluorescence in three independent patients showing expression of CD82, α-SMA, PDGFR-α and COL13A1 in human IPF and corresponding staining quantification. CD82$^+$ mean 85.6%, range, 67-97% and box bounds 84.5-89.5% representing first and third quantiles. CD82$^-$ mean 19.7%, range, 7-33% and box bounds 12.5-27.0% representing first and third quantiles. 2-sided unpaired *t*-test. Scale bar 50 µm (*n* = 3 IPF patients, 3 independent experiments). **e**, Microscopy images showing immunohistochemistry of CD82 and COL13A expression in human IPF with corresponding isotype control. Scale bar 40 µm.
